# Hipertensão Pulmonar na Síndrome do Ovário Policístico

**DOI:** 10.36660/abc.20210106

**Published:** 2021-04-08

**Affiliations:** 1 Clínica Mayo Divisão de Doenças Cardiovasculares Rochester EUA Divisão de Doenças Cardiovasculares, Clínica Mayo, Rochester - EUA

**Keywords:** Síndrome do Ovário Policístico, Hipertensão Pulmonar, Obesidade, Resistência à Insulina, Ecocardiografia Doppler/métodos, Índice de Massa Corpórea, Remodelação Ventricular

A Síndrome do Ovário Policístico (SOP) é uma síndrome endocrinológica complexa que se apresenta em mulheres com obesidade, resistência à insulina e anormalidades nos hormônios sexuais. É intrigante que na hipertensão pulmonar “idiopática”, também parece haver uma alta prevalência das mesmas características de obesidade, resistência à insulina e anormalidades nos hormônios sexuais.^1^^–^^3^ No entanto, apesar dessa sobreposição e do risco teórico de hipertensão pulmonar na SOP, pouco se sabe sobre a intersecção das duas condições. Dada a idade jovem das pacientes no momento do diagnóstico de SOP, somente comprometimentos leves têm sido consistentemente descritos na função cardíaca esquerda,^4^^,^^5^ com a doença cardiovascular evidente frequentemente se manifestando muitas décadas depois.^6^^,^^7^ (6,7). Mas, surpreendentemente, pouco se sabe sobre a remodelação cardíaca direita subclínica ou hipertensão pulmonar nessa condição.

Nesse contexto, Abacioglu et al.^8^ fornecem novas informações sobre a remodelação cardíaca estrutural em pacientes com SOP, com atenção especial à estrutura e função cardíacas direitas. Eles incluíram 44 pacientes com SOP e 60 controles pareados que foram submetidos a ecocardiografia abrangente e avaliação da resistência à insulina pelo modelo de avaliação da homeostase da resistência à insulina (HOMA-IR). Além das medidas da função diastólica cardíaca esquerda, os autores também realizaram o Doppler por onda de pulso da via de saída do ventrículo direito para estimar a rigidez arterial pulmonar e utilizaram a bem validada relação TAPSE/PSVD para quantificar o acoplamento VD-AP.

A coorte do estudo com SOP e os controles foram bem pareados em idade basal e índice de massa corporal (IMC) geral. O grupo com SOP era, em média, jovem (média de idade de 22 anos), com IMC médio normal (24,9 kg/m^2^) e ausência de outros fatores de risco cardiovascular, mas a resistência à insulina era pior no grupo com SOP de acordo com sua fisiopatologia subjacente. Em geral, não houve diferenças na disfunção sistólica ou diastólica no lado esquerdo pela ecocardiografia. Entretanto, a rigidez da artéria pulmonar, a função do lado direito do coração e o acoplamento VD-AP foram piores no grupo SOP. A rigidez da artéria pulmonar se correlacionou com a resistência à insulina e apresentou tendência a ser mais elevada em pacientes que não estavam em tratamento para a SOP.

Embora a amostra do estudo seja pequena, os grupos foram bem pareados na demografia geral, além da resistência à insulina, permitindo a avaliação de deficiências subclínicas secundárias à SOP. No entanto, as diferenças entre os grupos foram pequenas e a avaliação da progressão em longo prazo dessas mudanças no acoplamento VD-AP para determinar a significância clínica é necessária. Alterações nos hormônios sexuais também foram identificadas em pacientes com HAP ou SOP, e como esses influenciam, são desconhecidos no acoplamento VD-AP anormal nesta amostra.

Essas questões à parte, este estudo fornece informações importantes sobre o papel potencial da SOP na hipertensão pulmonar em mulheres. É notável que, apesar dos avanços modernos, uma grande proporção dos casos de HAP permanece idiopática com um efeito desproporcional nas mulheres. Dada a mudança dos dados demográficos da HAP para um fenótipo mais obeso nos tempos modernos,^9^ o fato de que a síndrome metabólica, a resistência à insulina e a obesidade podem estar contribuindo para a hipertensão pulmonar é uma questão de grande importância para a saúde pública. A adiposidade visceral, em particular, está mais fortemente ligada à resistência à insulina e pode ser acentuadamente diferente para o mesmo IMC e, preferencialmente, piora a hemodinâmica central em mulheres.^10^

Estudos futuros são necessários para verificar se as diferenças na adiposidade visceral na SOP podem ser subjacentes a algumas dessas alterações observadas no coração direito. A perda de peso em pacientes com sobrepeso, mesmo sem insuficiência cardíaca, pode melhorar as pressões da artéria pulmonar e a hemodinâmica central^11^ e, dado o papel central da obesidade em muitas pacientes com SOP, isso pode ter implicações terapêuticas importantes para a saúde cardiovascular em longo prazo.

Uma advertência importante para estudos ecocardiográficos como este, é a subestimação sistêmica da carga de doença cardíaca esquerda e insuficiência cardíaca precoce com fração de ejeção preservada que é cada vez mais reconhecida em indivíduos jovens com sobrepeso.^12^^–^^14^ Os parâmetros de ecocardiografia tradicionais não são sensíveis para remodelação cardíaca esquerda precoce e insuficiência cardíaca com fração de ejeção preservada.^14^^,^^15^ e se as pressões de enchimento do coração esquerdo forem maiores do que o esperado, isso pode contribuir para uma rigidez anormal da artéria pulmonar. O grupo de SOP neste estudo também foi um tanto atípico em relação ao fato de que o IMC médio era não de obesidade. Portanto, o achado de que a função diastólica do ventrículo esquerdo não estava prejudicada neste estudo pode não ser generalizável para outras coortes de SOP onde a remodelação subclínica do coração esquerdo foi relatada anteriormente.^10^ A obesidade está independentemente associada à remodelação cardíaca direita progressiva,^16^ acoplamento VD-AP anormal e juntamente com pressões de enchimento cardíacas esquerdas elevadas.^13^^,^^14^ Portanto, na SOP, a síndrome metabólica, a obesidade (12) e principalmente a adiposidade visceral^17^ associadas podem ter um efeito remodelador crônico no coração e predispor à insuficiência cardíaca com fração de ejeção preservada e hipertensão pulmonar associada no futuro. Dado o grande número de jovens afetadas por SOP, o estudo de Abacioglu et al.^8^ configura-se uma chamada urgente para uma investigação mais profunda sobre a relação entre SOP e o risco futuro de hipertensão pulmonar.
