# Supplementary material for: Hipertensão Pulmonar na Síndrome do Ovário Policístico
Source: Arq Bras Cardiol. 2021 Apr 8;116(4):812–3. [Article in Portuguese] doi: 10.36660/abc.20210106 (PMC8121395; doi:10.36660/abc.20210106)
Supplement: Translation

# Translation: Pulmonary Hypertension in Polycystic Ovarian Syndrome

**Affiliations:** 1 Rochester USA Divisão de Doenças Cardiovasculares, Clínica Mayo, Rochester - USA

**Keywords:** Polycystic Ovary Syndrome, Pulmonary Hypertension, Obesity, Insulin Resistance, Echocardiography/methods, Body Mass Index, Ventricular Remodeling

Polycystic Ovarian Syndrome (PCOS) is a complex endocrinological syndrome that presents in women with obesity, insulin resistance and sex hormone abnormalities. It is intriguing that in otherwise ‘idiopathic’ pulmonary hypertension, there also seems to be a high prevalence of the same features of obesity, insulin resistance, and sex hormone abnormalities.^1^^–^^3^ However, despite this overlap and a theoretical risk of pulmonary hypertension in PCOS, little is known about the intersection of the two conditions. Given the young age of patients at the time of PCOS diagnosis, only subtle impairments in left heart function have been consistently described,^4^^,^^5^ with overt cardiovascular disease often manifesting many decades later.^6^^,^^7^ But, surprisingly, little is known about subclinical right heart remodeling or pulmonary hypertension in this condition.

On this background, Abacioglu et al.^8^ provide novel information on cardiac structural remodeling in patients with PCOS, with careful attention to right heart structure and function. They included 44 patients with PCOS and 60 matched controls who underwent comprehensive echocardiography and insulin resistance assessment by the Homeostatic Model Assessment for Insulin Resistance (HOMA-IR). In addition to left heart measures of diastolic function, the authors also performed pulse wave Doppler of the right ventricular outflow tract to estimate pulmonary arterial stiffness, and used the well validated TAPSE/RVSP ratio to quantify RV-PA coupling.

The study cohort of PCOS and controls were well-matched regarding baseline age and overall body mass index (BMI). The PCOS group was on average young (mean age 22) with a normal mean BMI (24.9 kg/m^2^) and showed absence of other cardiovascular risk factors, but insulin resistance was worse in the PCOS group, consistent with their underlying pathophysiology. Overall, there were no differences in left-sided systolic or diastolic dysfunction by echocardiography. However, pulmonary artery stiffness, right-sided function and RV-PA coupling was worse in the PCOS group. Pulmonary artery stiffness correlated with insulin resistance and tended to be higher in patients who were not undergoing treatment for PCOS.

Although the study sample is small, the groups were well-matched in overall demographics apart from insulin resistance, allowing assessment of subclinical impairments secondary to PCOS. However, the differences between the groups were small and the evaluation of the long-term progression of these changes in RV-PA coupling to determine clinical significance is needed. Sex hormone alterations have also been identified in patients with either PAH or PCOS, and how these influence the abnormal RV-PA coupling in this sample is unknown.

These issues aside, this study provides important information on the potential role of PCOS in pulmonary hypertension in women. It is remarkable that despite modern advances, a large proportion of PAH cases remain idiopathic with a disproportionate effect on women. Given the shifting demographics of PAH to a more obese phenotype in modern times,^9^ the role that metabolic syndrome, insulin resistance and obesity may have in pulmonary hypertension is a question of great public health importance. Visceral adiposity in particular is more strongly linked with insulin resistance and can be markedly different for the same BMI and preferentially worsens central hemodynamics in women.^10^

Whether differences in visceral adiposity in PCOS may underlie some of the observed right heart changes requires future studies. Weight loss in overweight patients, even in those without heart failure, may improve pulmonary artery pressures and central hemodynamics,^11^ and given the central role of obesity in many patients with PCOS, this may have important therapeutic implications for long-term cardiovascular health.

An important caveat to echocardiographic studies such as this, is the systemic underestimation of the burden of left heart disease and early heart failure with preserved ejection fraction that is increasingly recognized in young overweight individuals.^12^^–^^14^ Traditional echocardiographic parameters are not sensitive for early left heart remodeling and heart failure with preserved ejection fraction,^14^^,^^15^ and if the left heart filling pressures are higher than expected, this can contribute to abnormal pulmonary artery stiffness. The PCOS group in this study was also somewhat atypical in the fact that the average BMI was that of non-obese individuals. Therefore, the finding that left ventricular diastolic function was not impaired in this study may not be generalizable to other PCOS cohorts where subclinical left heart remodeling has been previously reported.^10^ Obesity is independently associated with progressive right heart remodeling,^16^ abnormal RV-PA coupling, along with elevated left heart filling pressures.^13^^,^^14^ Therefore in PCOS, the associated metabolic syndrome, obesity (12) and particularly visceral adiposity^17^ may have a chronic remodeling effect on the heart and predispose to heart failure with preserved ejection fraction and associated pulmonary hypertension in the future. Given the large number of young individuals affected by PCOS, the study by Abacioglu et al.^8^ should be an urgent call for further investigation into the relationship between PCOS and the future risk of pulmonary hypertension.
